# Book Reviews

**Published:** 1915-09

**Authors:** 


### BOOK REVIEWS

Books mentioned may be inspected at and ordered through this office. So far as possible, books received in any month will be reviewed in the issue of the second month following.

---

**Anti-Prohibition Manual.** Published by the National Wholesale Liquor Dealers' Assn. of America, 301 United Bank Bldg., Cincinnati.

A pamphlet containing statistics of the workings of prohibition, of interest in itself and presenting arguments against interference with personal liberty.

---

**The Medical Clinics of Chicago.** Volume I. Number I. (July 1915). Octavo of 208 pages, 37 illustrations. Philadelphia and London: W. B. Saunders Company, 1915. Published Bi-Monthly. Price per year: Paper, $8.00. Cloth $12.00.

The first issue is a volume of 208 pages, illustrated. A number of physicians and surgeons are represented and the topics are not limited to any one branch of medicine.

**Exercise in Education and Medicine.** By R. Tait McKenzie, A. B., M. D., Professor of Physical Education, and Director of the Department, University of Pennsylvania. Octavo of 585 pages, with 478 illustrations. Philadelphia and London: W. B. Saunders Company, 1915. Cloth, $4.00 net; Half Morocco, $5.50 net.

This book treats of the training of athletes, of children in ordinary schools and in those for the blind, deaf-mute and other defectives, of the availability of exercise for the relief of deformities, and in the treatment of visceral diseases. The dangers of over-strain are carefully considered. Special methods of exercise such as massage are also described in detail.

---

**A Manual of the Practice of Medicine.** By A. A. Stevens, A. M., M. D., Professor of Therapeutics and Clinical Medicine in the Woman's Medical College of Pennsylvania, Lecturer on Medicine in the University of Pennsylvania. Tenth Edition, Revised. 12mo of 629 pages, illustrated. Philadelphia and London: W. B. Saunders Company, 1915. Flexible Leather, $2.50 net.

---

This is one of the very best works on practice—we were about to say ''on a scale intermediate between the quiz compend and the monumental treatise''—but will omit the qualification. That it should reach a tenth edition is good evidence of its appreciation by the profession. Its small size is somewhat deceptive for the publishers' art has resulted in a degree of compactness which gives, at first glance, the impression of much less matter than is actually presented.

---

**Quiz Compends—Chemistry.** Inorganic and Organic, including Urinary Analysis, by Henry Leffmann, A. M., M. D., Philadelphia. P. Blakiston's Son & Co., Philadelphia. 241 pages, illustrated, $1.00.

This is the sixth edition of the favorite, brown covered series. It is unnecessary to speak of the value of these works for medical students nor to point out the fallacies of the argument against condensed manuals that will prepare students for the examinations required of him. So long as the examination system is retained, such books must exist. Nor do we need to remind the graduate physician, required to pass an examination for a license in another state, of the help rendered by such books. What we want particularly to mention is that such books have a value for one with no such ulterior motives. We would advise the physician who is rusty on chemistry, to buy this book and read it carefully, thus refreshing his memory and bringing his information up to date.

---

The following interesting reports have been received at this office and are acknowledged with thanks. They may be referred to here or, later at the Grosvenor Library.

**Report of the N. Y. State Veterinary College** for the year 1913-14.

**Year Book of the Pilcher Hospital** for the period April 1-Dec. 31, 1914.

**Municipal Ordinances, Rules and Regulations Pertaining to Public Health.** Reprint No. 230 from Public Health Reports.

**State Laws and Regulations Pertaining to Public Health.** Reprint No. 200 from Public Health Reports.

**International Health Commission,** Rockefeller Foundation, first annual report, June 27, 1913-Dec. 31, 1914 (sic). This deals with the British West Indies, Egypt, Ceylon, Federal Malay States, Phillipines, Costa Rica, etc., the information having been obtained by personal representatives of the foundation on a tour.

**Annual Report of the Department of Health,** Buffalo, for 1914. As this publication has been distributed to the profession, we merely state that it is worth careful study, not only because it justifies a considerable amount of pride in a municipal department of the highest efficiency according to present standards but because the intelligent co-operation and support of the profession can aid greatly in securing further advance.

---

**The Practical Medicine Series,** under general editorial charge of Charles L. Mix, A. M., M. D., Chicago, published by the Year Book Publishers, Chicago, the price of the annual series of ten volumes being $10.00.

Vol. 3, Eye, Ear, Nose and Throat, edited by Casey A. Wood, C. M., M. D., D. C. L., Albert H. Andrews, M. D. and Wm. L. Ballenger, M. D. 384 pages, illustrated, $1.50 separately.

Vol. 4, Gynaecology, edited by E. C. Dudley, A. M., M. D. and Herbert M. Stowe, M. D. 234 pages, illustrated, $1.35 separately.

This series has become the standard review of current literature.

